# Phytochemicals targeting glycolysis in colorectal cancer therapy: effects and mechanisms of action

**DOI:** 10.3389/fphar.2023.1257450

**Published:** 2023-08-24

**Authors:** Lu Zhan, Fangting Su, Qiang Li, Yueqiang Wen, Feng Wei, Zhelin He, Xiaoyan Chen, Xiang Yin, Jian Wang, Yilin Cai, Yuxia Gong, Yu Chen, Xiao Ma, Jinhao Zeng

**Affiliations:** ^1^ Department of Oncology, Hospital of Chengdu University of Traditional Chinese Medicine, Chengdu, China; ^2^ School of Basic Medicine, Chengdu University of Traditional Chinese Medicine, Chengdu, China; ^3^ Guang’an Hospital of Traditional Chinese Medicine, Guang’an, China; ^4^ State Key Laboratory of Southwestern Chinese Medicine Resources, School of Pharmacy, Chengdu University of Traditional Chinese Medicine, Chengdu, China; ^5^ Department of Gastroenterology, Hospital of Chengdu University of Traditional Chinese Medicine, Chengdu, China; ^6^ TCM Regulating Metabolic Diseases Key Laboratory of Sichuan Province, Hospital of Chengdu University of Traditional Chinese Medicine, Chengdu, China

**Keywords:** colorectal cancer, warburg effect, glycolysis, phytochemicals, molecular pathways

## Abstract

Colorectal cancer (CRC) is the third most common malignant tumor in the world, and it is prone to recurrence and metastasis during treatment. Aerobic glycolysis is one of the main characteristics of tumor cell metabolism in CRC. Tumor cells rely on glycolysis to rapidly consume glucose and to obtain more lactate and intermediate macromolecular products so as to maintain growth and proliferation. The regulation of the CRC glycolysis pathway is closely associated with several signal transduction pathways and transcription factors including phosphatidylinositol 3-kinases/protein kinase B/mammalian target of rapamycin (PI3K/AKT/mTOR), adenosine 5′-monophosphate (AMP)-activated protein kinase (AMPK), hypoxia-inducible factor-1 (HIF-1), myc, and p53. Targeting the glycolytic pathway has become one of the key research aspects in CRC therapy. Many phytochemicals were shown to exert anti-CRC activity by targeting the glycolytic pathway. Here, we review the effects and mechanisms of phytochemicals on CRC glycolytic pathways, providing a new method of drug development.

## 1 Introduction

Colorectal cancer (CRC) ranks third among the globally most prevalent cancers, in terms of incidence and mortality (Sheng et al., 2020). The morbidity and mortality of CRC are consistently increasing, which poses a serious threat to the general population’s health (Jian et al., 2020). After initial CRC diagnosis, 20% of the patients develop metastatic disease, and an additional 25% of the patients with initially localized disease will subsequently develop metastasis (Biller and Schrag, 2021). At present, surgical resection and chemo-radiotherapy are the most common treatment options for CRC patients (Liang et al., 2019). Surgery is the only curative treatment of CRC, however, even after radical surgery, a high recurrence rate remains (van der Stok et al., 2017). Further, there is considerable resistance to CRC to chemotherapy (Van der Jeught et al., 2018). Therefore, the current treatment of CRC is in urgent need of novel and more effective avenues.

The Warburg effect of cancer cells dictates that even under aerobic conditions, cancer cells require glycolysis for energy, which is a hallmark of tumor metabolism (Salimian Rizi et al., 2015). Normal cells generate energy primarily through mitochondrial oxidative phosphorylation, and glycolysis is increased only under hypoxia (Leung et al., 2015). The glucose uptake by cancer cells is markedly higher than that of normal cells, and most of the pyruvate produced is converted to lactic acid through lactate dehydrogenase (LDH). Compared with 36 ATP molecules produced by oxidative metabolism, glycolysis yields only 2 ATP plus 2 lactic acid molecules from 1 molecule of glucose (Gatenby and Gillies, 2004). Although the efficiency of ATP production through glycolysis is lower, the ATP generation rate through glycolysis is nearly 100-fold faster, compared to oxidative phosphorylation (Zhou et al., 2012). Further, rapid aerobic glycolysis produces many intermediates that are conducive to the growth of tumor cells, and these intermediates maintain the proliferation, invasion, and metastasis of tumor cells (Lunt and Vander Heiden, 2011). The metabolic pathway of the Warburg effect has become the focus of tumor treatment in recent years, and its related targeted pathways will be used as an important approach for researching treatment options for colorectal malignancies.

Phytochemicals are an important source of medicines, and plant drugs still play an important role in the treatment of diseases in developing countries (Tamene and Endale, 2019). Despite the rapid development of synthetic drugs, natural compounds remain one of the main sources of drugs (Kazantseva et al., 2022). Approximately 25% of anti-cancer drugs are derived from phytochemicals and contain one or more plant-active ingredients (Kopaskova et al., 2011). At present, many anticancer drugs are derived from phytochemicals, such as vinblastine, paclitaxel, and camptothecin (Wang H. et al., 2018). With the wide application of phytochemicals in cancer treatment and synthetic drug development becoming more difficult, research on anticancer drugs is focused on the development and utilization of phytochemicals and their active substances (Tong et al., 2019), and phytochemicals targeting tumor metabolism have been extensively studied. Moreover, the synergistic effects of phytochemical drugs and anticancer drugs have been attracting attention, as a combination of the two drugs can inhibit the recurrence and metastasis of cancer and reduce the resistance of tumor cells to chemotherapy (Lee et al., 2021). Therefore, it is vital to accelerate the development and utilization of phytochemical drugs for cancer treatment.

Here, we review research on phytochemicals and their active components acting on the glycolysis pathway of CRC cells, and we discuss the molecular mechanisms of phytochemicals targeting the glycolysis pathway to treat CRC. This review will provide a clue for further research on the treatment of CRC targeting the glycolysis pathway.

## 2 Aerobic glycolysis in CRC

**FIGURE 1:**
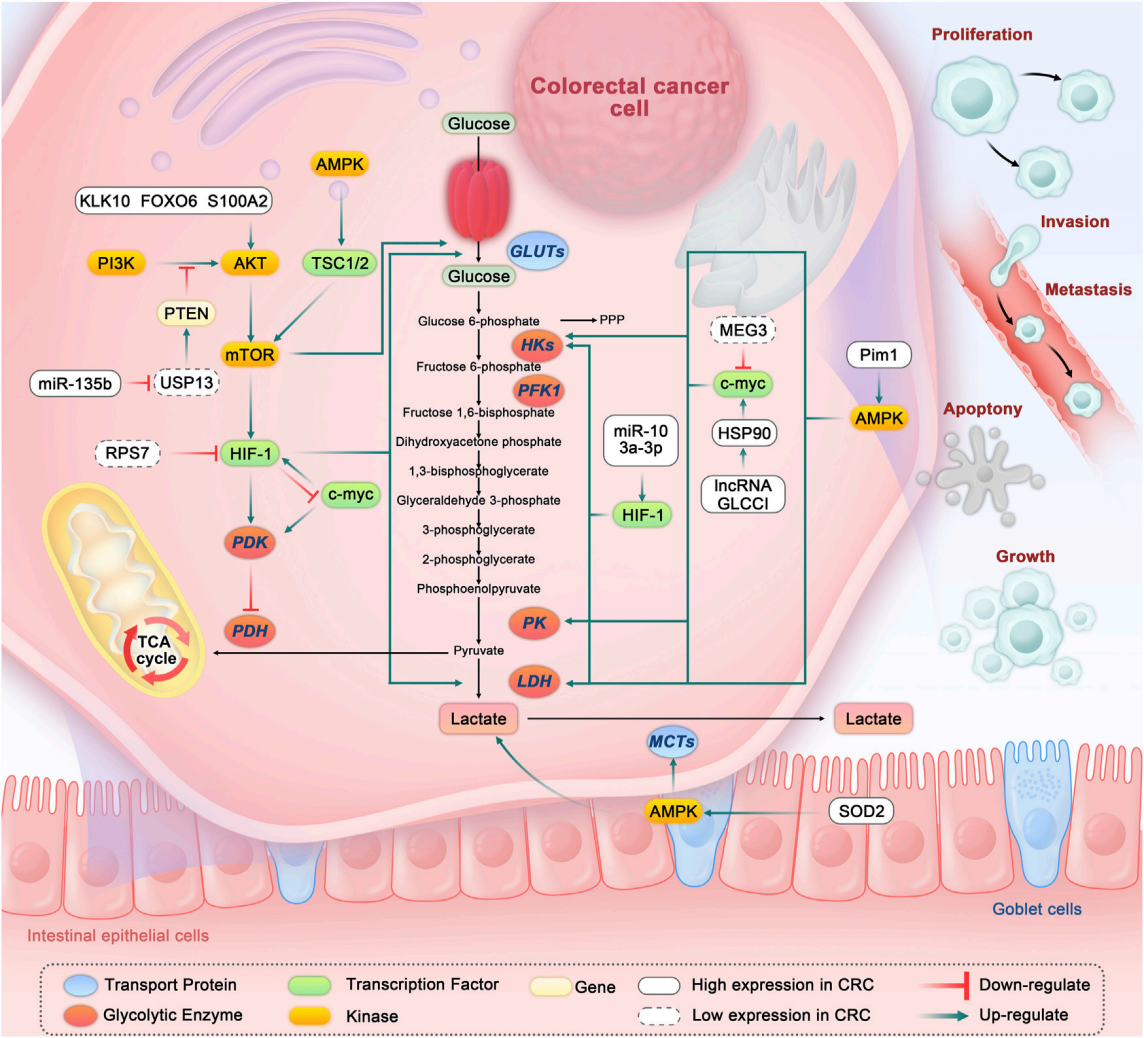
Glycolysis in colorectal cancer cells and the related regulatory pathways. Abbreviation: GLUTs, Glucose transporters; HKs, Hexokinases; PFK1, Phosphofructokinase-1; PK, Pyruvate kinase; LDH, Lactate dehydrogenase; PDK, Pyruvate dehydrogenase kinase; PDH, Pyruvate dehydrogenase; AMPK, Adenosine 5′-monophosphate-activated protein kinase; TSC1/2, Tuberous sclerosis complex1/2; AKT, Protein kinase B; mTOR, Mammalian target of rapamycin; HIF-1, Hypoxia-inducible factor-1; PI3K, Phosphatidylinositol 3-kinases; PTEN, Phosphatase and tensin homolog; USP13, Ubiquitin-specific peptidase 13; RPS7, ribosomal protein S7; TCA, Tricarboxylic acid; PPP, Pentose phosphate pathway; HSP90, Heat shock protein 90; MCTs, Monocarboxylic acid transporters; MEG3, lncRNA maternally expressed gene 3; SOD2, Superoxide dismutase 2.

Substantial evidence suggests that metabolic enzymes and transporters associated with the glycolytic pathway are highly expressed in CRC, and these changes may be related to the regulation of several signaling pathways and transcription factors (As shown in Figure 1).

### 2.1 Upregulation of the glycolytic pathways in CRC

The glycolytic metabolism of CRC cells is closely associated with particular transporters and key enzymes of the glycolytic pathway. Glucose metabolism in cells is mediated by glucose transporters (GLUTs), which occur in the cell membrane. There are 14 subtypes of the GLUT gene (Ancey et al., 2018), of which GLUT1, as one of the most intensively studied membrane transporters, is a key speed-limiting factor of glucose metabolism (Thorens and Mueckler, 2010). This transporter is highly expressed in various cancer tissues, including CRC (Younes et al., 1995; Baer et al., 1997; Mori et al., 2007; Feng et al., 2017), and it has been confirmed to be related to the progress and metastasis of CRC (Younes et al., 1996; Haber et al., 1998). Hexokinase (HK) is a catalytic enzyme that converts glucose into glucose-6-phosphate and is the first rate-limiting enzyme in the glycolysis pathway. There are four types of HK isoenzymes, among which HK2 is abnormally highly expressed in the metabolic pathway of tumor cells, and its expression is closely associated with CRC proliferation and metastasis (Shinohara et al., 1994; Wilson, 2003; Katagiri et al., 2017; Huang et al., 2021). Phosphofructokinase-1, as a catalytic enzyme for the synthesis of 1,6-fructose-diphosphate from fructose-6-phosphate, is one of the key rate-limiting enzymes in the glycolytic pathway. Genes encoding phosphofructokinase-1 activity are highly expressed in CRC (Houddane et al., 2017). The key regulatory enzyme of the pyruvate kinase (PK) glycolysis pathway is responsible for the dephosphorylation of phosphoenolpyruvate to pyruvate and ATP. There are four PK isoenzymes, of which PKM2 is highly expressed in many malignant tumors, including colorectal tumors (Wong et al., 2014; Cui and Shi, 2015; Han et al., 2016). PKM2 maintains the high glycolysis rate of tumor cells and regulates the proliferation of tumor cells (Yu et al., 2015). In the process of tumor glycolysis, most pyruvate is converted into lactic acid by LDH (Li et al., 2020b). LDHA is highly expressed in colorectal and other malignant tumors and is closely related to tumor proliferation and metastasis (Wang et al., 2015; Xian et al., 2015; Cui et al., 2017; Zhao et al., 2017).

Monocarboxylic acid transporters (MCTs) are lactic acid transporters located on the cell membrane, which are responsible for transporting intracellular lactic acid to the exterior of the cell to maintain the pH in tumor cells; further, they are highly expressed in primary CRC and are associated with CRC metastasis and prognosis (Martins et al., 2016). Pyruvate can be converted to acetyl coenzyme A (CoA) under the action of the pyruvate dehydrogenase complex, and enter mitochondria for metabolism through the oxidative phosphorylation pathway (Saunier et al., 2016). Pyruvate dehydrogenase complex acts as the gatekeeper of the oxidative phosphorylation pathway, and the respective enzymes are inhibited in CRC to maintain the high glycolysis rate of CRC and its proliferation and invasion (Hamabe et al., 2014; Ho and Coomber, 2015). Therefore, targeting pyruvate dehydrogenase complex may be a new direction for CRC treatment. The above glycolytic metabolic pathways are abnormally expressed in CRC cells and have exacerbating effects on the occurrence and development of CRC.

### 2.2 Regulatory mechanisms of glycolytic pathways in CRC

Enzymes and transporters related to the CRC glycolysis pathway are abnormally expressed by different molecular regulatory mechanisms, thus leading to the occurrence and development of CRC.

#### 2.2.1 PI3K/AKT/mTOR

PI3K/AKT/mTOR signaling pathway is a cellular signal transduction pathway, which affects cell growth, proliferation, angiogenesis, and other processes (Coutte et al., 2012; He et al., 2022; Hu et al., 2023). Activation of the PI3K/AKT signal pathway plays an important role in maintaining the glucose metabolism of cells, as it can increase glucose intake by increasing the expression of GLUT and upregulate the expression of glycolysis-related enzymes to promote cell glycolysis (Chang et al., 2015; Prabakaran et al., 2018). AKT is frequently overexpressed in most CRC, whereas phosphatase and tensin homolog (PTEN) expression is lost (Robey and Hay, 2009). As a tumor suppressor gene, PTEN negatively regulates the PI3K/AKT/mTOR pathway (Hollander et al., 2011), and deletion of PTEN can specifically increase the protein level of HIF-1α through PI3K signaling (Jiang et al., 2001), mTOR is closely related to cell growth and mTOR expression is regulated by a variety of factors, including the energy regulator AMPK. mTOR can also regulate the expression of HIF-1α by sensing the intracellular level of hypoxia (Martin and Hall, 2005).

Kallikrein-related peptidase, forkhead box class O6, and S100 calcium-binding protein A2 are highly expressed in CRC and play an activating role in glycolysis by activating PI3K/AKT/mTOR pathway and up-regulating GLUT1 expression (Li et al., 2019; Li C. et al., 2020; Wei et al., 2020). MicroRNAs are non-coding RNA molecules that play a central part in cell differentiation, proliferation, and survival (Rupaimoole and Slack, 2017). MiR-135b is highly expressed in CRC cells, which can activate PI3K pathway and promote glycolysis of CRC cells by down-regulating the expression of ubiquitin-specific peptidase 13 and reducing the stability of PETN (Xiang et al., 2015).

#### 2.2.2 AMPK

AMPK is a conservative Ser/Thr protein kinase, which regulates cell metabolism and maintains the dynamic balance of cell metabolism, thus it is referred to as a regulator of energy homeostasis (Faubert et al., 2013; Narayanankutty, 2019). AMPK is activated by sensing the increase of AMP and ATP (Tokunaga et al., 2019). Once activated, AMPK will immediately block the energy consumption process of cells, and turn to increase the ATP decomposition process (Wang and Guan, 2009). AMPK is an important kinase of cell metabolism and has been widely studied in many metabolic diseases (Towler and Hardie, 2007).

Superoxide dismutase 2 (SOD2) is a key antioxidant enzyme that is highly expressed in CRC. The reduction of SOD2 can significantly downregulate AMPK phosphorylation and the expression of MCT4 and L-lactate and ultimately inhibit the migration and glycolytic metabolism of CRC cells (Zhou C. et al., 2020). Pim1 is an oncogene promoting the growth and metastasis of CRC, which is highly expressed in CRC cells, and it has been found that pim1 can upregulate the expression of HK2 and LDHA in CRC cells, which may be related to the activation of the AMPK pathway under glucose deprivation (Zhang et al., 2018).

#### 2.2.3 HIF-1

Compared with normal cells, the oxygen consumption of tumor cells is markedly increased to maintain cancer cells rapid proliferation, thus leading to relative hypoxia in the local microenvironment (Georges et al., 2018). HIF plays a critical role in driving tumor growth, invasion, and metastasis and is found to be highly expressed in most solid tumors (Kumar and Gabrilovich, 2014; Jiang et al., 2020). HIF - 1 has been demonstrated to participate in regulating key transcription factors of EMT and indirectly promotes EMT by Notch, TGF-β, Wnt, and Hedgehog signal pathways, thereby promoting tumor invasive metastasis (Rankin and Giaccia, 2016). HIF-1 can also induce the expression of the epidermal growth factor receptor (EGFR), transforming growth factor-β, insulin-like growth factor 2 to promote tumor angiogenesis (Bui et al., 2022). HIF has been confirmed to be involved in the glycolytic pathway of tumor cells, and it can directly upregulate the expression of glycolysis-related transporters and key enzymes such as GLUT, PFK, LDH, HK, etc. (Song et al., 2016). At the same time, HIF can also be activated by PI3K/AKT (Yeh et al., 2018), YAP/TAZ (Simula et al., 2022), hippo/YAP1 (Sun et al., 2020) and other signaling pathways to further promote the glycolytic process of tumor cells.

*miR-103a-3p* is an oncogene that is highly expressed in CRC tissues and cell lines and promotes the glycolysis pathway and proliferation, invasion, and migration of CRC cells by up-regulating the transcription of HK2, LDHA, and PKM1 through the Hippo/YAP1/HIF1α axis (Sun et al., 2020). The ribosomal protein S7 gene (RPS7), as a tumor suppressor gene plays a role in inhibiting glycolysis of CRC cells by inhibiting the expression of HIF-1α, GLUT4, and LDHB (Zhang et al., 2016).

#### 2.2.4 Myc

Myc is highly overexpressed in human cancers, thus providing energy for tumor growth and proliferation and the synthesis of substrates required for the respective metabolism pathways (Dang et al., 2006; Miller et al., 2012). c-myc can directly upregulate the expression of LDHA and promote the conversion of pyruvate to lactic acid, and it can upregulate the expression of enzymes related to glycolytic pathways, such as HK2 and phosphoinositide-dependent protein kinase 1, and promote the glycolytic metabolism of cells (Dang et al., 2006; Miller et al., 2012). Myc is inhibited by HIF-1α, however, the two show synergistic effects in regulating the expression of glycolysis-related enzymes including HK2 and PDK1 (Yeung et al., 2008).

Long non-coding RNAs (lncRNAs) are commonly involved in tumor metabolic rewiring and immune cell infiltration and functioning (Zhang Y. et al., 2021). The lncRNA maternally expressed gene 3 (MEG3) reduces glycolytic levels in CRC cells by degrading the expression of c-myc, including down-regulating the expressions of glycolysis-related enzymes such as LDHA, PKM2, and HK2 (Zuo et al., 2020). Thus, MEG3 expression is inhibited in CRC cells (Zuo et al., 2020). Glycolysis-associated lncRNA of colorectal cancer (lncRNA GLCC1) interacts with heat shock protein90 (HSP90) to stabilize c-myc transcription and thus target c-myc-mediated LDHA expression (Tang et al., 2019).

## 3 Targeting glycolysis in colorectal cancer therapy

Increasing evidence suggests that metabolic reprogramming is closely related to the occurrence and development of most tumors, including CRC (Nenkov et al., 2021). Cancer cells show a significant increase in aerobic glycolysis in their metabolism, making them potentially more susceptible to inhibition of glycolytic pathways than normal cells. Numerous studies on CRC metabolic pathways attempted to identify new therapeutic directions, including the glycolysis pathway, and the drug-targeting glycolysis pathway is an attractive strategy for CRC therapy.

lncRNAs play an important role in the epigenetic regulation of cancer progression by regulating cell function and development (Zheng et al., 2021). Some lncRNAs are highly expressed in CRC, and they exert anti-tumor effects by regulating the glycolytic pathway (Wang Y. et al., 2019; Liu et al., 2020; Li C. et al., 2021). Chemotherapeutic resistance is a major challenge in CRC treatment, and reprogramming of glucose metabolism is also associated with chemical drug resistance, and high glycolysis levels found in human CRC drug-resistant models (Wang T. et al., 2018). Targeted inhibition of the glycolytic pathway can increase the sensitivity of CRC cells to chemotherapy drugs (Cao et al., 2018; An and Ha, 2022). 5-FU is an important drug in first-line chemotherapy for CRC. In CRC cells resistant to 5-FU, glucose uptake and lactic acid production increase, and glycolysis-related enzymes and GLUT1 and MCT1/4 are significantly upregulated, which may be related to the activation of PI3K/AKT and the Wnt/β-catenin signaling pathway by upregulation of HIF-1α (Dong et al., 2022). The onset of CRC is closely associated with intestinal environment disorders caused by lifestyle and dietary habits, thus probiotics show promising prospects in the treatment of CRC (Donovan et al., 2017; Eslami et al., 2020). Butyrate, a short-chain fatty acid produced by bacterial fermentation of dietary fibers in the colon, was the first probiotic identified as a histone deacetylase inhibitor, which may be related to the inhibition of the Warburg effect in CRC (Hamer et al., 2008; Donohoe et al., 2012; Eslami et al., 2020).

In conclusion, the glycolytic pathway can affect the occurrence and development of CRC through various processes. Therefore, studying the mechanism of CRC and reprogramming glucose metabolism in tumor cells may provide a new research strategy for CRC treatment (Vasaikar et al., 2019).

## 4 Methods

We conducted a comprehensive literature search using the Web of Science, PubMed, Embase, SpringerLink, ScienceDirect, and EBSCO databases from the beginning of the database to 30 September 2022 and collated and analyzed the literature for this review. The literature search was conducted by two authors independently and separately to minimize potential oversights. This review evaluates and summarizes all previous scientific studies on the effects of phytochemicals on CRC glycolysis pathways. In PubMed, we used MeSH search terms such as “colorectal neoplasm,” “colorectal cancer,” “glycolysis,” “Embden Meyerhof pathway,” “Warburg effect,” “phytochemicals” and “natural product.” We excluded non-experimental articles and repeated articles in the search process.

**FIGURE 2:**
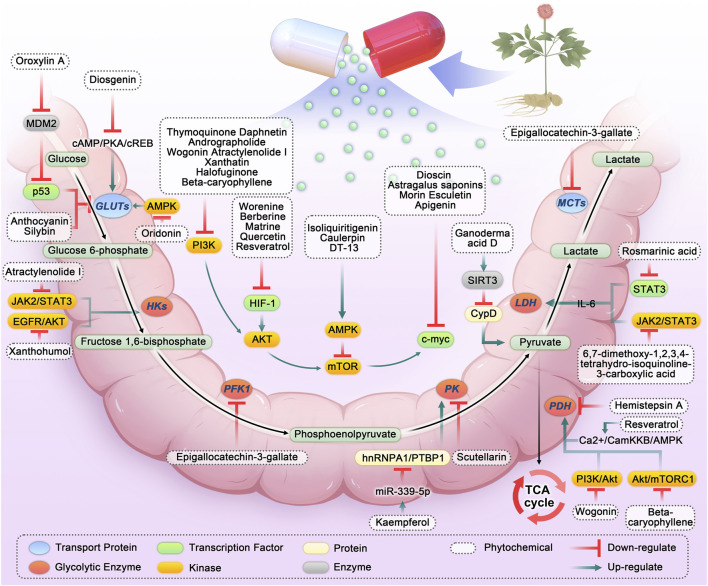
Phytochemicals targeting glycolysis in colorectal cancer. Abbreviation: HKs, Hexokinases; PFK1, Phosphofructokinase-1; PK, Pyruvate kinase; LDH, Lactate dehydrogenase; MCTs, Monocarboxylic acid transporters; PDH, Pyruvate dehydrogenase; TCA, Tricarboxylic acid; IL-6, Interleukin 6; SIRT3, Sirtuin-3; JAK2/STAT3, Janus kinase 2/signal transducer and activator of transcription 3; PI3K, Phosphatidylinositol 3-kinases; AKT, Protein kinase B; HIF-1, Hypoxia-inducible factor-1; AMPK, Adenosine 5′-monophosphate-activated protein kinase; hnRNPA1, Heterogeneous nuclear ribonucleoprotein A1; PTBP1, polypyrimidine tract-binding protein 1; PTEN, Phosphatase and tensin homolog.

**FIGURE 3:**
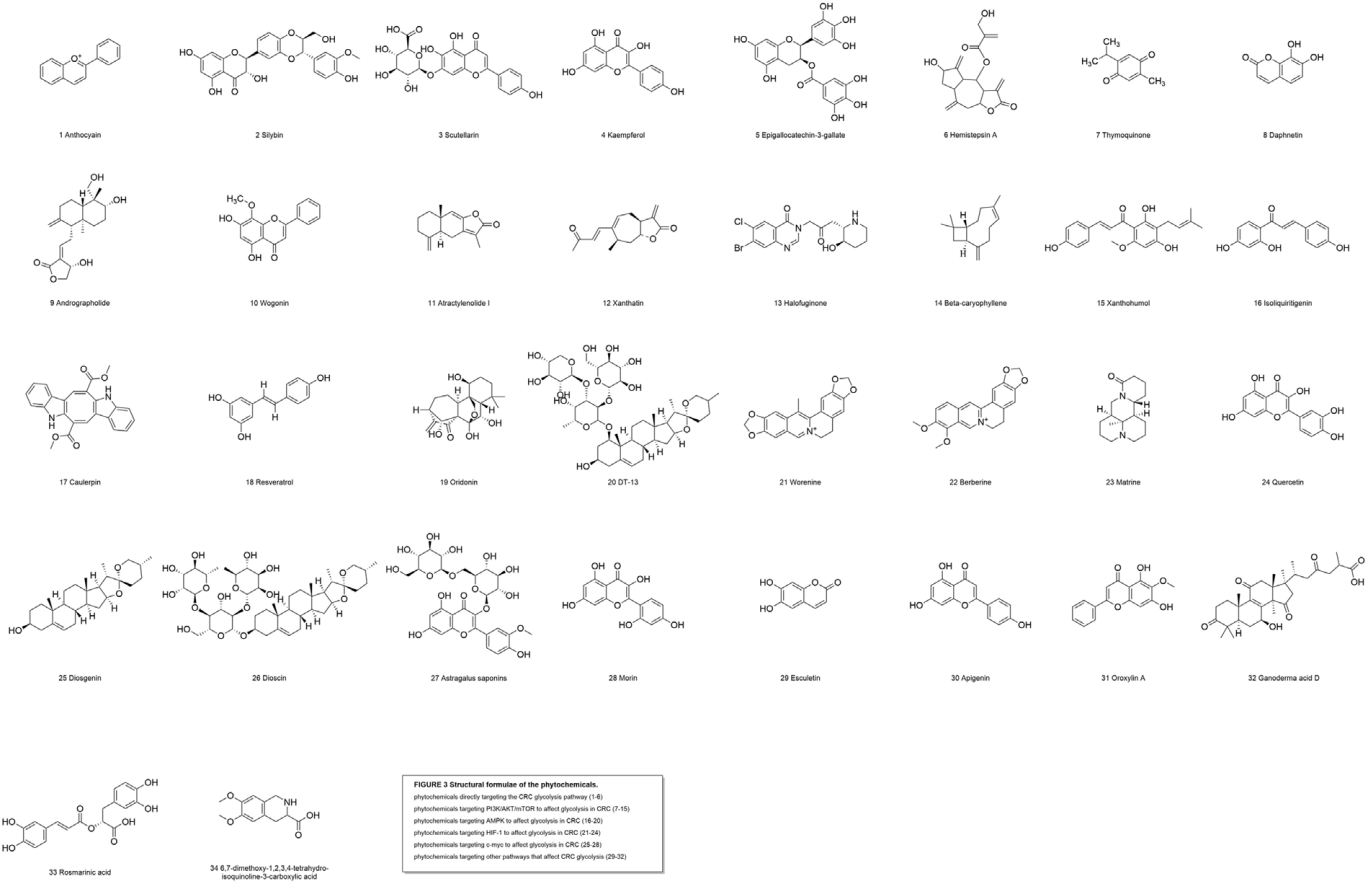
Structural formulae of the phytochemicals.

Through screening and summarizing plant chemicals targeting the CRC glycolytic pathway, we found that numerous *in vitro* and *in vivo* studies confirmed that plant chemicals can target the CRC glycolytic pathway and exert anti-cancer effects (As shown in Figures 2, 3; Table 1). Here, the targets affecting the glycolytic pathway are divided into 1) phytochemicals directly targeting the CRC glycolysis pathway; 2) phytochemicals targeting PI3K/AKT/mTOR to affect glycolysis in CRC; 3) phytochemicals targeting AMPK to affect glycolysis in CRC; 4) phytochemicals targeting HIF-1 to affect glycolysis in CRC; 5) phytochemicals targeting c-myc to affect glycolysis in CRC; 6) phytochemicals targeting other pathways that affect CRC glycolysis.

**TABLE 1:** Phytochemicals targeting glycolysis for colorectal cancer therapy.

Signal pathways and transcription factors in glycolysis	Compound	Source	Experimental model		Efficacy	Mechanism	Experimental dosage	References
			*In vitro*	*In vivo*				
GLUT1	Anthocyanin (Flavonoids)	*grape, mulberry, and raspberry*	MC38	—	Disturbtion of glucose transport; inhibition of energy metabolism	↓GLUT1	500,1000, 2000 µM (24 h)	Jing et al. (2020)
	Silybin (Flavonoids)	*Silybum marianum* (L.) *Gaertner*	LoVo	—	Downregulation of the glucose uptake	↓GLUT1	5, 10, 50 µM (24/48/72 h)	Catanzaro et al. (2018)
PKM2	Scutellarin (Flavonoids)	*Erigeron breviscapus* (Vant.) Hand.-Mazz.	SW480 HT29	OR-SW480 cells mouse model	Induction of apoptosis; reduction of ATP product; inhibition of glycolysis	↓PKM2	2 µM 10 ㎍/kg	Sun et al. (2021)
	Kaempferol (Flavonoids)	*Acacia nilotica* (L.) Delile*, Aloe vera* (L.) Burm.f.*, Crocus sativus* L*.*	HCT116 DLD1	—	Inhibition of glycolysis and ATP product; induction of apoptosis	↑miR-339-5p; ↓hnRNPA1; ↓PTBP1; ↓PKM2	0, 50, 100 µM (48 h)	Wu et al. (2021)
MCT4	Epigallocatechin-3-gallate (Polyphenols)	*green tea*	HCT116; HT29	—	Inhibition of the proliferation, migration and metastasis; Inhibition of glycolysis	↓PFK; ↓MCT4	25, 50, 100 µM	Chen et al. (2022)
PDH	Hemistepsin A (Sesquiterpene lactone)	*Hemistepta lyrata*	DLD1 CT26	CT26 cells xenograft tumors	Inhibition of cell growth; inhibition of glycolysis; induction of apoptosis	↓PDK1; ↓PDHA1	0,5,7.5.10 µM(4 h)	Jin et al. (2020)
							1 mg/kg, 10 mg/kg	
PI3K/AKT/mTOR	Thymoquinone (Quinones)	*Nigella sativa*	HCT116; SW480	—	Inhibition of proliferation and migration; induction of apoptosis; inhibition of glycolysis and ATP product	↓PI3K; ↓AKT; ↓HK2	21.71 µM, 20.53 µM	Karim et al. (2022)
	Daphnetin (Coumarins)	*Daphne Korean Nakai*	SW480 HT29	—	Inhibition of proliferation and migration; induction of apoptosis; inhibition of glycolysis	↓PI3K; ↓AKT; ↓HK2; ↓GLUT1	0, 25, 50 µM (24 h)	He et al. (2018)
	Andrographolide (Diterpenoids)	*Andrographis paniculata*	HCT116	—	inhibition of glycolysis and ATP product	↓HK2; ↓PFK1; ↓GLUT1; ↓PI3K; ↓AKT; ↓mTOR	20 µM	Li et al. (2020c)
	Wogonin (Flavonoids)	*Scutellaria baicalensis*	HCT116	HCT116 cells xenograft mice	inhibition of glycolysis and ATP product; Inhibition of tumor growth	↓PI3K/AKT; ↓HIF-1α; ↓HK2; ↓PDHK1; ↓LDHA; ↓GLUT1	20, 40, 60, 80, 100 µM(24 h) 30, 60 mg/kg; 0, 10, 20, 40 µM	Wang et al. (2014)
								Zhao et al. (2018)
	Atractylenolide I (Sesquiterpene lactones)	*Atractylodis macrocephalus*	HCT116 Colo205	HCT116 xenograft nude mice	Disruption of glucose metabolism; induction of apoptosis; inhibition of invasion and glycolysis; Inhibition of tumor growth	↓AKT/mTOR→↓GLUT1; ↓LDH; ↓HK2; ↓PKM2; ↓JAK2/STAT3 → ↓HK2	0, 80, 150, 200 μM (24 h)	Wang et al. (2020b)
							25, 75 mg/kg; 0, 100, 200 µM (24 h) 50 mg/kg	Li et al. (2020d)
	Xanthatin (Sesquiterpene lactones)	*Xanthium strumarium* L*.*	HT-29 HCT-116	—	inhibition of glycolysis and ATP product	↓mTOR→↓GLUT1; ↓HK1; ↓MCT4	0, 20, 30, 40 µM (24 h)	Li et al. (2022a)
	Halofuginone (Alkaloids)	*Dichroa febrifuga*	SW480 HCT116	HCT116 xenograft nude mice	induction of apoptosis; inhibition of glycolysis; Inhibition of tumor growth	↓AKT/mTORC1→↓P70S6K; ↑4EBP1→↓ HK2; ↓GLUT1	0, 5, 10, 20 nM	Chen et al. (2015)
							0.1 mg/kg	
	Beta-caryophyllene (Sesquiterpenes)	*Syzygium aromaticum*	CT26	diabetic Balb/c mice	Inhibition of proliferation and glycolysis; induction of apoptosis	↓p-AKT; ↓p-mTOR; ↓c-myc; ↓PDK1; ↓LDHA	50 µM	Zhou et al. (2018)
							200 mg/kg	
	Xanthohumol (Polyphenol)	*Humulus lupulus* L.	HCT116	HT29 and HCT116 cells xenotransplantation mice	Inhibition of proliferation and glycolysis; induction of apoptosis; Inhibition of tumor growth	↓EGFR/AKT→↓ERK1/2→↓HK2	0,2,4,8 µM	Liu et al. (2019)
			SW620 HT29				10 mg/kg	
AMPK	Isoliquiritigenin (Flavonoids)	*Glycyrrhiza uralensis, Mongolian glycyrrhiza, Glycyrrhiza glabra*	HCT116	—	Inhibition of proliferation, glycolysis and ATP product; induction of apoptosis	↑AMPK→↓AKT/mTOR→↓ENO1; ↓ALDOA; ↓LDHA; ↓MCT4; ↓c-myc; ↓HIF-1α	12.5, 25, 50, 100 µM	Wang et al. (2020a)
	Caulerpin (Alkaloids)	*Caulerpa racemosa, C. serrulata*	LoVo	SW480 xenograft nude mice	Disturbtion of glycolysis Delay the tumor growth	↑AMPK→↓mTORC1; ↓GLUT1	5,10,20 µM 30 mg/kg	Yu et al. (2017)
			SW480					
	Resveratrol (Polyphenols)	*pomegranates, berries, peanuts,* and *red wine*	Caco2 HCT116 CT26 HT29	CT26 tumor bearing mice	inhibition of glycolysis; Inhibition of tumor growth	↑Ca2+/CamKKB/AMPK→↑PDH; ↓HIF-1α	10 µM (48 h); 50 μM; 50 µM	Saunier et al. (2017)
								Jung et al. (2015)
								Jung et al. (2013)
	Oridonin (Diterpenoid)	*Rabdosia rubescens*	SW480	SW480 xenograft tumors	Inhibition of proliferation and glycolysis; Inhibition of tumor growth	↓AMPK; ↓GLUT1; ↓MCT1	5,10,15,20.25 µM	Yao et al. (2017)
	DT-13 (Saponins)	*Liriope muscari* (Decne.) Bailey	HT29 HCT116 HCT15 COLO205	HCT-15 orthotopic nude mice	Inhibition of proliferation and glycolysis; Inhibition of tumor growth	↑AMPK→↓mTOR→↓GLUT1; ↓HK2; ↓PFKM; ↓LDHA	2.5, 5, 10 μM (72 h)	Wei et al. (2019)
							0.625, 1.25, 2.5 mg/kg	
HIF-1	Worenine (Alkaloids)	*Coptis chinensis*	HCT116 SW620	—	inhibition of glycolysis	↓HIF-1α→↓GLUT3; ↓HK2; ↓PFK-L; ↓PKM2; ↓LDHA; ↓PKM	50,10,20 μM (24 h)	Ji et al. (2021)
	Berberine (Alkaloids)	*Rhizoma coptidis*	HCT116 KM12C	—	inhibition of glycolysis	↓mTOR; ↓HIF-1α→↓GLUT1; ↓LDHA; ↓HK2	0,6.25,12.5, 25,50,100 µM	Mao et al. (2018)
	Matrine (Alkaloids)	*Sophora flavescens*	HCT116 SW620	—	inhibition of glycolysis	↓HIF-1α→↓GLUT1; ↓HK2; ↓LDHA	0,2,4,6,8 µM	Hong et al. (2019)
	Quercetin (Flavonoids)	*apples, cabbage, cauliflower, and berries*	HCT116	F344 rats	induction of apoptosis; inhibition of glycolysis; upregulation of the mitochondrial fatty acid degradation	↓AMPK→↓HIF-1α→↓VEGF; ↓GLUT1 ↓F-1,6-BP; ↓GAPDH; ↓ENO1; ↓PK ↑3-hydroxy-3-methylglutaryl-CoA synthase 2; ↑acetyl-CoA acyltransferase 1; ↑acyl-CoA dehydrogenase short-chain	100 µM; 10 g/kg	Kim et al. (2012)
								Dihal et al. (2008).
c-myc	Diosgenin (Saponins)	*legumes, fenugreek, yams*	SW1116 RKO	SW1116 cells xenograft mice	Inhibition of proliferation, invasion and metastasis; induction of apoptosis; inhibition of glycolysis; Inhibition of tumor growth	↓cAMP/PKA/CREB→↓GLUT3; ↓GLUT2; ↓PC	20 µM	Li et al. (2021b)
	Dioscin (Saponins)	*Dioscoreae rhizome, Paridis rhizome*	HT29 HCT116 SW480 SW620	HCT116 and HT29 xenograft mice; HT29 and SW620 xenograft mice	Inhibition of proliferation and glycolysis; Inhibition of tumor growth	↓c-myc; ↓Skp2; ↓Cdh1→↓HK2	0, 1, 2, 5 μM, 5 mg/kg; 0,1,2,4 μM, 10 mg/kg	Zhou et al. (2020b), Wu et al. (2020)
	Astragalus saponins (Saponins)	*Astragalus membranaceus*	SW620	DSS-induced colitis mouse model	induction of apoptosis; Inhibition of proliferation and glycolysis	↓c-myc→↓HK2; ↓LDHA	50 μg/mL (0, 12, 24 and 48 h)	Guo et al. (2019)
							0.1 mg/g	
	Morin (Flavonoids)	*mulberry figs, fustic*	SW480	DMH induce colon cancer model	Reduce the incidence of tumors; inhibition of glycolysis and ATP production; induction of apoptosis	↓β-cateinin/c-myc; ↓GLUT1; ↓HK2; ↓PKM2; ↓LDHA	50 μg/kg	Sharma et al. (2017), Sithara et al. (2017)
							150, 200, and 250 μM (48 h)	
	Esculetin (Coumarins)	*Fraxinus rhynchophylla* Hance	—	DMH induce colon cancer model	Reduce the incidence of tumors; downregulate the expression of proto-oncogenes; affect tumor metabolism	↓β-cateinin/c-myc	50 μg/kg	Sharma et al. (2017)
						↓GLUT1; ↓HK2; ↓PKM2; ↓LDHA		
	Apigenin (Flavonoids)	*parsley, celery, chamomile*	HCT116 HT29 DLD1	—	inhibition of the cell survival and colony formation; inhibition of glycolysis	↓β-catenin/c-myc/polypyrimidine tract binding protein 1; ↓PKM2	0, 20, 40 µM	Shan et al. (2017)
p53	Oroxylin A (Flavonoids)	*Scutellaria baicalensis Georgi.*	HCT116	HCT116 xenograft mice	inhibition of glycolysis; Inhibition of tumor growth	↑p53; ↑TIGAR; ↑SIRT3 ↓PGM; ↓GLUT4; ↓MDM2	200 μM (48 h)	Zhao et al. (2015)
							100 mg/kg	
SIRT3/Cypd	Ganoderma acid D (Triterpenoids)	*Ganoderma lucidum*	HT29 SW620	—	inhibition of glycolysis	↓CypD→↑SIRT3	0,50,100,200 μmol/L	Liu et al. (2018)
IL/STAT3	Rosmarinic acid (Phenylpropanoids)	*Rosmarinus officinalis* L. (Lamiaceae)	HCT8 HCT116	—	inhibition of glycolysis	↓miR-155-5p→↓IL-6/STAT3→↓LDH; ↓HIF-1α	075,150 μmol/L (24 h)	Xu et al. (2016)
	6,7-dimethoxy-1,2,3,4-tetrahydro-isoquinoline-3-carboxylic acid (Alkaloids)	*Mucuna pruriens*	—	DMH induce CRC rats	inhibition of glycolysis	↓IL-6→↓JAK2/STAT3→↓LDH	25 mg/kg	Mishra et al. (2018)

## 5 Phytochemicals targeting signal pathways and transcription factors in the CRC glycolysis pathway

### 5.1 Phytochemicals directly targeting CRC glycolysis pathway

#### 5.1.1 Anthocyanin

Anthocyanin (ANC) is a flavonoid that is common in plants (McGhie and Walton, 2007). And after glycosylation, ANC glycosides can be rapidly absorbed by the human body (McGhie et al., 2003). Cyanidin-3-glucoside, a member of the anthocyanin family, is found in purple or red vegetables and fruits (Shan et al., 2021). A comparative study using cyanidin-3-glucoside and its anthocyanidin aglycone showed that cyanidin-3-glucoside significantly inhibited the expression of GLUT1 in MC38 (mouse colon cancer cells), and it can disturb glucose transport, inhibit energy metabolism, and cause mitochondrial damage and apoptosis in CRC cells (Jing et al., 2020).

#### 5.1.2 Silybin

Silybin is the major component of silymarin, a flavonolignan mixture extracted from the fruits of *Silybum marianum* (L.) Gaertner displays antioxidant, anti-inflammatory, immunomodulatory and hepatoprotective properties (Chambers et al., 2017). Compared with doxorubicin-sensitive LoVo cells (LoVo WT), doxorubicin-resistant LoVo cells (LoVo DOX) show higher mRNA and protein expression levels of glycolysis-related enzymes, such as GLUT1 and MCT4 (Catanzaro et al., 2018). Further, it also shows a tendency for metabolic transfer from the oxidative phosphorylation pathway to the glycolysis pathway. After treatment of cells with silybin 10–50 μM, glucose uptake and GLUT1 expression is downregulated in sensitive and drug-resistant LoVo cells in silybin 10 μM, and silybin 50 μM resulted effective only in resistant cells (Catanzaro et al., 2018).

#### 5.1.3 Scutellarin

Scutellarin is a flavonoid drug derived from the plant *Erigeron breviscapus* (Vant.) Hand.-Mazz. (Chen et al., 2006; Dong and Qu, 2022). *Erigeron breviscapus* (Vant.) Hand.-Mazz. is a Chinese herbal medicine that was first recorded in South Yunnan Materia Medica with multiple pharmacological effects and clinical applications, such as detoxification, inflammation reduction and pain relief (Chen et al., 2006; Dong and Qu, 2022). Studies have found that Oxaliplatin-resistant CRC cells (OR-SW480 and OR-HT29) exhibit higher glycolysis rate and high mRNA and protein expression of PKM2, while scutellarin can inhibit the glucose metabolism rate and ATP production of cells by decreasing the PKM2 expression of OR-SW480 and OR-HT29 cells, leading to mitochondrial dysfunction and finally inducing cell apoptosis (Sun et al., 2021). This previous study established an *in vivo* model using OR-SW480 cells injected into 4-week-old female immunodeficient nude BALB/c mice, showing that 10 mg/kg scutellarin reversed the drug resistance of oxaliplatin in the OR-SW480 mouse model, increased apoptosis induced by oxaliplatin, and significantly reduced protein expression of PKM2 and ATP production in the tumor (Sun et al., 2021).

#### 5.1.4 Kaempferol

Kaempferol is a flavonoid compound that occurs in many plants such as *Acacia nilotica* (L.) Delile, *Aloe vera* (L.) Burm.f., and *Crocus sativus* L, and has been shown to be cardioprotective, anti-inflammatory, antidiabetic, antioxidant, antitumor, and have anticancer activities (Devi et al., 2015; Imran et al., 2019). Studies have shown that kaempferol can exert anticancer effects by inducing apoptosis of cancer cells, causing cell cycle arrest and autophagy (Wu et al., 2018). Studies have shown that after Kaempferol treatment, the cell cycle of HCT116 and DLD1 cells is delayed, and apoptosis is upregulated (Wu et al., 2021). In addition, glucose consumption, lactic acid production, and ATP level exhibit a downward trend. Kaempferol can also upregulate the expression of miR-339-5p in HCT116 and DLD1 cells, reducing the expression of hnRNPA1 and PTBP1, leading to the downregulation of PKM2 expression, thereby inhibiting glycolysis of CRC (Wu et al., 2021).

#### 5.1.5 Epigallocatechin-3-gallate

Green tea, which originated in China, is one of the most popular drinks in the world and was used in traditional Chinese medicine to treat many illnesses (Akbarialiabad et al., 2021). Epigallocatechin-3-gallate (EGCG) is mainly sourced from green tea (Nagle et al., 2006). EGCG inhibits the proliferation, migration, and metastasis of HCT116 and HT29 cells (Chen et al., 2022). In addition, in cancer-associated fibroblasts (CAFs) co-cultured with HCT116 and HT29 cells, 50 μM EGCG significantly decreased lactate production, PFK, and MCT4 expression (Chen et al., 2022). After further silencing the expression of MCT4, it was found that the above inhibitory effect of EGCG was reversed. Therefore, it is speculated that EGCG may inhibit the glycolysis of CAFs through MCT4.

#### 5.1.6 Hemistepsin A

*Hemisteptia lyrata* is a kind of Chinese herb, its leaves and flowers are used for the treatment of sore throat and treatment of tumor (Jang et al., 1999). Hemistepsin A (HsA) is a sesquiterpene lactone isolated from *Hemistepta lyrata* Bunge, which exerts various pharmacological effects including anti-hepatotoxic, anti-inflammatory, and anti-cancer activities (Park et al., 2020). Treatment of various CRC cell lines with HsA showed that HsA had significant cytotoxicity, and significantly inhibited cell growth, but had no significant effect on normal cells (Jin et al., 2020). Further, after treatment with HsA, the extracellular acidification rate (ECAR) of DLD1 and CT26 cells decreased in a dose-dependent manner. Further study of its internal mechanism found that HsA inhibited the activity of PDK1 by interfering with the interaction between PDK1 protein and lipoamide binding domains of PDH-E2 (L1 and L2), reduced the phosphorylation of PDHA1, and thus inhibited the formation of lactic acid. *In vivo*, experiments showed that HsA (1, 10 mg/kg/day) treated for 10d inhibited the growth of BALB/c mice bearing CT26 cells in a dose-dependent manner, upregulated the expression of apoptosis-related genes, and reduced the phosphorylation of PDHA1, which was consistent with the results of *in vitro* experiments (Jin et al., 2020).

### 5.2 Phytochemicals targeting PI3K/AKT/mTOR to affect glycolysis in CRC

#### 5.2.1 Thymoquinone

*Nigella sativa* is a flowering plant that belongs to the Ranunculaceae family, native to south and southwest Asia, and is used to treat a variety of ailments such as diarrhea, asthma, headaches, cough, eczema, and more (Ciesielska-Figlon et al., 2023). Thymoquinone is a bioactive constituent derived from the seeds of *N. sativa* and has shown significant anticancer activity in various tumors (Phua et al., 2021). Thymoquinone significantly inhibits the proliferation, invasion, and metastasis of HCT116 and SW480 cells, induces cell apoptosis, and downregulates the lactic acid production, glucose uptake, and ATP levels of tumor cells (Karim et al., 2022). Furthermore, it exerts anti-CRC effects by inhibiting HK2 expression under the PI3K/AKT pathway, thus affecting the glycolysis pathway of CRC cells (Karim et al., 2022).

#### 5.2.2 Daphnetin

Daphnetin, a coumarin derivative, was first isolated from *Daphne Korean Nakai*, and it exerts various pharmacological activities such as anticoagulation, anti-inflammatory, heart protection, and anti-cancer effects (Hang et al., 2022; Javed et al., 2022). After daphnetin treatment for 24 h, the proliferation and migration of SW480 and HT29 cells are inhibited, apoptosis is increased, glucose uptake and lactic acid production are inhibited, and the protein expression levels of HK2 and GLUT1 are downregulated (He et al., 2018). In addition, the phosphorylated expression of PI3K and AKT is significantly downregulated in SW480 cells treated with daphnetin, LY294002 (a PI3K/AKT inhibitor) could enhance the inhibitory effect of daphnetin on the glycolysis pathway in SW480 cells. Thus daphnetin may exert anti-cancer effects by targeting the PI3K/AKT pathway to affect glycolysis in CRC cells (He et al., 2018).

#### 5.2.3 Andrographolide

*Andrographis paniculata* belongs to the genus Andrographis, widely distributed in Asian countries such as India, China, and Malaysia, and has been often used to treat inflammatory diseases (Li et al., 2022c). Andrographolide is the principal active ingredient of *Andrographis paniculate* extract, which has shown great potential in the treatment of a variety of inflammatory diseases as well as tumors (Zhang H. et al., 2021; Qu et al., 2022). Andrographolide not only inhibits glucose uptake and lactic acid production in HCT116 cells, decreases ATP levels, and inhibits the expression of glycolysis proteins and enzymes, such as PFK1, HK2, and GLUT1, but also inhibits the phosphorylation of the PI3K/AKT/mTOR pathway (Li et al., 2020c). Further, IGF-1 (a PI3K activator) can reverse the downregulated expression of PI3K, p-AKT, and p-mTOR by andrographolide, and andrographolide shows similar inhibitory effects on HCT116 as LY294002 (a PI3K inhibitor). Therefore, andrographolide may inhibit CRC by targeting PI3K/AKT signal pathway. Interestingly, andrographolide may also improve the radio sensitivity of HCT116 cells through the PI3K/AKT pathway (Li et al., 2020c).

#### 5.2.4 Wogonin

*Scutellaria Baicalensis*, belonging to the Lamiaceae family, is a commonly used Chinese herb for clearing heat and detoxification (Yang et al., 2022). Wogonin is a flavonoid from *Scutellaria baicalensis*, which has anti-tumor, anti-inflammatory, antiviral, and other pharmacological effects (Banik et al., 2022). Wogonin at 20, 40, 60, 80, and 100 mM for 24 h can downregulate the expressions of HK2, PDHK1, and LDHA by inhibiting the transcriptional activity of PI3K/AKT and the expression of HIF-1α, thus affecting the glucose uptake and lactic acid production of HCT116 cells under hypoxia (Wang et al., 2014). Meanwhile, an *in vivo* study showed that 30 and 60 mg/kg wogonin could significantly reduce the tumor weight of male BALB/c nude mice, and decreased the expression of HIF-1α, glycolysis-related proteins, and PI3K/AKT (Wang et al., 2014). In another study, wogonin inhibited the survival of HCT116 cells and the glycolysis of HCT116 expressing wild type in a dose-dependent manner (Zhao et al., 2018). Wogonin increased the protein level as well as mRNA level of p53 and TIGAR in HCT116 cells, and decreased the protein and mRNA level of PGM, HK2, GLUT1, PDHK1, and LDHA, however, in p53-null and mt-p53 HCT-116 cells the expression and transcription of the glycolysis regulator barely changed. The experiment showed that the inhibitory effect of wogonin on cancer cell glycolysis was dependent on wt-p53. (Zhao et al., 2018).

#### 5.2.5 Atractylenolide I

*Atractylodes macrocephala* Koidz., which has been used for thousands of years in China, is mainly used to treat gastrointestinal diseases such as loss of appetite, abdominal distension and diarrhea, has the functions of invigorating the spleen for strengthening the stomach, and dispelling dampness for diuresis (Li et al., 2022b). Atractylenolide I (AT-I) is a sesquiterpenoid lactone derivative of *Atractylodis macrocephalus* (Zhu et al., 2018). AT-I downregulates the phosphorylation of proteins related to the AKT/mTOR pathway and the protein expression of GLUT1, LDH, HK2, and PKM2 in a dose-dependent manner in HCT116 and Colo205 cells, thus disrupting the glucose metabolism of colorectal tumors, followed by induction of apoptosis and reduced cell invasion (Wang K. et al., 2020). In Balb/c nude mice were xenografted with HCT116 cells treated with 25 and 75 mg/kg AT-I, the tumor growth and the expression of glycolysis-related proteins were significantly inhibited, it is consistent with the *in vitro* experimental results (Wang K. et al., 2020). In addition, AT-I downregulates the expression of HK2 in HCT116 cells by inhibiting the JAK2/STAT3 pathway and reduces glucose consumption and lactate production in HCT116 cells, thus exerting anti-CRC effects (Li Y. et al., 2020). Similar results were also shown *in vivo* using an HCT116 transplanted mouse model treatment with AT-I at 50 mg/kg/day for 3 weeks. JAK2/STAT3, as a signaling network pathway, is responsible for transducing the signals conveyed by many cytokines from the cell membrane to the nucleus, and extensive studies have confirmed that JAK2/STAT3 plays an important role in the occurrence and development of tumors and drug resistance (Mengie Ayele et al., 2022).

#### 5.2.6 Xanthatin

*Xanthium strumarium L* (Asteraceae) is a commonly traditional Chinese herb have been applied for treating various diseases, including rhinitis, headache, arthritis, etc. (Fan et al., 2019). Xanthatin is a bioactive sesquiterpene lactone isolated from *X. strumarium* L. (Fan et al., 2019), which suppresses the growth of many tumors, such as lung cancer (Tao et al., 2016), glioma (Ma et al., 2020), and melanoma (Li et al., 2013). In addition, xanthatin downregulates ATP levels, lactic acid production, and glucose uptake in HCT116 and HT29 cells by inhibiting the mTOR pathway, and the mRNA and protein levels expression of GLUT1, MCT4 and the protein levels expression of HK1 is also inhibited (Li L. et al., 2022). Interestingly, elevated oxygen consumption rates (OCR) and downregulated succinic acid expression were observed in xanthatin-treated HT29 cells, which may be related to xanthatin-induced oxidative phosphorylation (Li L. et al., 2022). These results suggest that xanthatin may inhibit the glycolytic pathway of CRC cells by targeting the mTOR signaling pathway, in compensation for the activation of oxidative phosphorylation metabolism in cells, thereby causing mitochondrial dysfunction and ultimately inhibiting the growth and metastasis of CRC cells.

#### 5.2.7 Halofuginone

Chang Shan (*Dichroa febrifuga Lour*) is a traditional Chinese medicine as a treatment for malaria, dating back about 2000 years of history (Zhou et al., 2013). Halofuginone is a natural quinazolinone alkaloid extracted from the plant *Dichroa febrifuga* (Jain et al., 2021). Halofuginone is less toxic to normal cells, but it significantly inhibits the growth of CRC and induces reactive oxygen species (ROS) production and cell apoptosis in a dose-dependent manner (Chen et al., 2015). Moreover, halofuginone downregulates the phosphorylation of AKT, mTORC1, and downstream p70S6K in SW480 and HCT116 cells, while the phosphorylation of 4EBP1 is increased in a dose-dependent manner. P70S6K is one of the effectors of mTORC1, which can activate the pentose phosphate pathway, and 4EBP1 inhibits glucose uptake and glycolysis. Halofuginone also significantly decreases the protein expression of HK2 and GLUT1 and the production of tricarboxylic acid cycle intermediates, indicating that halofuginone inhibits the glycolysis pathway of CRC, which may be mediated by the AKT/mTORC1 signaling pathway. A vivo experiment showed that halofuginone at 0.1 mg/kg for 14 days downregulated the tumor weight and AKT/mTORC1 expression in female BALB/c nude mice xenotransplanted with HCT116 cells, which was consistent with the results of an *in vitro* experiment (Chen et al., 2015).

#### 5.2.8 Beta-caryophyllene

Beta-caryophyllene (BCP) is a bicyclic sesquiterpene that is a common constituent of essential oils, such as clove oil obtained from the dried flower-buds of *Syzygium aromaticum* (Machado et al., 2018). Arginine-specific-mono-ADP-ribosyltransfer 1 (ART1) is highly expressed in CRC and inhibits CRC proliferation, invasion, and metastasis (Yang et al., 2016). The glycolytic pathway is upregulated in ART1-expressing CT26 cells, while BCP can inhibit the expression of ART1-influenced glycolysis-related expression, such that of p-AKT, p-mTOR, c-Myc, PDK1 and LDHA, and it reduces lactate concentrations and ATP levels in CT26 cells, as shown using *in vivo* and *in vitro* models (Zhou et al., 2018) This suggests that BCP may inhibit ART1-induced glycolysis through the AKT/mTOR pathway, and it may play a role in inhibiting CRC cell proliferation and inducing apoptosis.

#### 5.2.9 Xanthohumol

Xanthohumol is a natural polyphenol chalcone from flowers of the common hop (*Humulus lupulus* L.) (Vicente de Andrade Silva et al., 2022). Xanthohumol has a variety of pharmacological activities, including antioxidant, anti-inflammatory, and anti-tumor effects (Jiang et al., 2018). The proliferation of HCT116, SW620, and HT29 cells is significantly inhibited and apoptosis is increased after treatment with xanthohumol (Liu et al., 2019); at the same time, glucose consumption, lactic acid production, and HK2 expression in cells are inhibited, and cell pH is decreased. In addition, xanthohumol inhibits the epidermal growth factor receptor (EGFR) and AKT signaling in a dose-dependent manner, and active Akt, Myr-Akt1 can rescued xanthohumol-induced HK2 suppression (Liu et al., 2019). Therefore, xanthohumol may downregulate HK2 expression by inhibiting the activation of the EGFR/AKT pathway, thus affecting the glycolytic pathway and inducing apoptosis in CRC cells. After xenotransplantation of HT29 and HCT116 cells into female athymic nude mice, the tumor weight of 10 mg/kg every 2 days in xanthohumol-treated mice was significantly reduced, and expression of Ki-67, p-AKT, and HK2 was downregulated, which was consistent with the results of *in vitro* experiment (Liu et al., 2019).

### 5.3 Phytochemicals targeting AMPK to affect glycolysis in CRC

#### 5.3.1 Isoliquiritigenin

Isoliquiritigenin (ISL) is one of the bioactive ingredients isolated from the roots of plants including *Glycyrrhiza uralensis*, *Mongolian glycyrrhiza*, and *Glycyrrhiza glabra* (Peng et al., 2015). ISL has a variety of biological activities, such as anti-inflammatory, antioxidant, nerve protection, and anti-tumor growth effects (Wang et al., 2021). ISL and ISL-loaded nanoparticles (ISL-NLs) inhibit proliferation, increase apoptosis, inhibit glucose uptake and lactate production, downregulate OCR and ATP levels, and decrease basal ECAR and maximum ECAR in HCT116 cells (Wang G. et al., 2020). Further, the respective study showed that the cell membrane potential was decreased, ROS levels were significantly increased, and the mRNA and protein levels of molecules enolase 1 (ENO1), aldolase, fructose-bisphosphate A (ALDOA), LDHA, MCT4, c-myc, and HIF-1α were also significantly decreased. The authors found that ISL and ISL-NLS could though activate AMPK and downregulate the expression of AKT and mTOR in HCT116 cells, thereby affecting cellular glucose metabolism. Compared with ISL, ISL-NLs have a stronger inhibitory effect on CRC (Wang G. et al., 2020).

#### 5.3.2 Caulerpin

Caulerpin is a bis-indole alkaloid, which has been isolated from *Caulerpa racemosa* and *C. serrulata* (Canché Chay et al., 2014). It has been proven to have anti-diabetic, anti-inflammatory, anti-tumor, anti-tuberculosis, and antibacterial effects (Lunagariya et al., 2019). A previous study found that caulerpin regulation of glucose metabolism in CRC cells depended on treatment time (Yu et al., 2017). After 8 h of caulerpin treatment, glucose metabolism in CRC cells was upregulated; GLUT1 was downregulated in LoVo and SW480 cells after 48 h; and p-AMPK increased during the first 30 min and decreased after 60 min. The downstream targets of mTORC1 4E-BP1 and S6 were also found to be inhibited. The above results may be related to the inhibition of ROS by caulerpin, resulting in reduced intracellular ATP levels and activation of the energy sensor AMPK. Once activated, AMPK promotes glycolysis to compensate for the loss of ATP. However, long-term activation of AMPK by caulerpin disrupts glycolysis and glucose metabolism in colorectal cells, ultimately leading to cell death (Yu et al., 2017). In mice transplanted with SW480, caulerpin (30 mg/kg) slowed tumor growth.

#### 5.3.3 Resveratrol

Resveratrol is a natural polyphenol occurring in a wide variety of fruits and vegetables, including peanuts, pistachios, and grapes (Ren et al., 2021), and it has biological activities such as antioxidation and antiproliferation effects (Jang et al., 1997). Studies have shown that in Caco^2^ and HCT116 cells, resveratrol by targeting the Ca^2+^/CamKKB/AMPK pathway, activates PDH, increasing the oxidative capacity of colorectal cancer cells, reducing glycolysis, and changing the metabolic pattern of tumor cells (Saunier et al., 2017). Resveratrol-loaded polymeric nanoparticles suppress glucose metabolism and tumor growth of CT26 cells *in vitro* and *in vivo* (Jung et al., 2015). In addition, resveratrol can also reduce F-FDG uptake and glycolytic metabolism in HT29 cells by downregulating HIF-1α (Jung et al., 2013).

#### 5.3.4 Oridonin

*Rabdosia rubescens* is a Chinese medicine to treat sore throat, gingivitis, and rheumatoid arthritis and is widely distributed in China (Abdullah et al., 2021). Oridonin, which is purified from *R. rubescens*, is an active diterpenoid compound with significant anticancer activity (Fujita et al., 1976; Yang et al., 2017). The inhibition of cell growth and apoptosis induction refers to various cancers, including prostate cancer, non-small cell lung cancer, and glioblastoma (Ikezoe et al., 2003). Oridonin not only showed anti-CRC cell activity *in vivo* and *in vitro* but also related to p53. This study further confirmed that oridonin could reduce glucose consumption and extracellular lactate concentration, down-regulating the mRNA and protein expression of GLUT1 and MCT1 in SW480 cells, thereby affecting cancer cell metabolism, and similar results were observed in SW480 xenograft BALB/c nude mice (Yao et al., 2017). However, interestingly, the increased ATP level in the CRC cells treated with oridonin was found, which may be related to the deactivation of AMPK, downregulate the expression of GLUT1 caused by oridonin, metabolic disorder of CRC cells, and thus induced autophagy (Yao et al., 2017).

#### 5.3.5 DT-13

Ophiopogonis Radix (Maidong in Chinese), the root of Ophiopogon japonicus, is widely used as a medicine in East Asia, with functions such as nourishing yin, promoting fluid production, and moistening the lungs (Chen et al., 2016). DT-13 is a saponin monomer of *Liriope muscari* (Decne.) Bailey has significant anti-tumor, anti-inflammatory, and heart protection effects (Zhang et al., 2015; Khan et al., 2018). DT-13 can inhibit the growth of HT29, HCT116, HCT15, and COLO205 cells (Wei et al., 2019). DT13 significantly inhibited glucose uptake, lactate production, and the expression of GLUT1 in HCT15 and HT29 cells, and also downregulated glycolysis-related mRNA and protein expressions such as HK2, PFK, and LDHA. Blocking GLUT1 attenuated the effects of DT13 on glucose uptake and cell proliferation in CRC cells (Wei et al., 2019). The *in vivo* study showed that DT-13 at dosages of 0.625, 1.25, and 2.5 mg/kg not only reduced tumor size and weight but also downregulated GLUT1 expression in HCT-15 orthotopic nude mice. Meanwhile, this study also found that DT13 could inhibit the proliferation of CRC cells by activating AMPK and inhibiting mTOR and its downstream phosphorylation. Treatment with an AMPK inhibitor (Compound C) alleviated the proliferation inhibition of DT13 (Wei et al., 2019).

### 5.4 Phytochemicals targeting HIF-1 to affect glycolysis in CRC

#### 5.4.1 Worenine

*Coptis Chinensis* (Huanglian in Chinese), a famous traditional herbal medicine used for clearing heat and detoxification, is widely used to treat inflammatory and other diseases (Wang J. et al., 2019; Yang et al., 2021). Worenine is one of the bioactive components in the dried rhizomes of *Coptis Chinensis* (Meng et al., 2018), and it inhibits cell viability and induces cell cycle arrest of HCT116 and SW620 cells *in vitro* (Ji et al., 2021). Worenine-treated HCT116 and SW620 cells, the production of lactic acid and the uptake and consumption of glucose is significantly inhibited, the protein and mRNA levels of GLUT3, HK2, PFK-L, PKM2, and LDHA in HCT116 cells are reduced, and PKM activity is also knockdown. The above changes were achieved by reducing the level of HIF-1α, desferrioxamine (stabilize HIF-1α) treatment for HCT116 cells reversed worenine-induced effects on the Warburg effect (Ji et al., 2021).

#### 5.4.2 Berberine

Berberine is a botanical alkaloid from the Ranunculaceae and Papaveraceae plant families (Mao et al., 2018). It is the active component of the Chinese medicine *Rhizoma coptidis* (Xiong et al., 2022), and it has shown anticancer activity in a variety of cancers, such as breast cancer (Zhong et al., 2022), lung cancer (Achi et al., 2022), gastric cancer (Liu et al., 2022), liver cancer (Wang et al., 2010), CRC (Sun Q. et al., 2022). Berberine significantly inhibits the growth and glucose uptake of HCT116 and KM12C cells in a dose-dependent manner and inhibited the mRNA levels of GLUT1, LDHA, and HK2. Further investigation of the underlying mechanism revealed that berberine inhibited glucose metabolism by suppressing mTOR-dependent HIF-1α protein synthesis in CRC cells (Mao et al., 2018).

#### 5.4.3 Matrine

Sophora flavescens (Fabaceae), which has the effect of killing insects and dispelling dampness, has a long history of use for thousands of years (Sun P. et al., 2022). Matrine is a natural quinoline alkaloid that occurs in the traditional Chinese medicine *Sophora flavescens* (Lai et al., 2003; Zhang et al., 2020a). A study on the anti-tumor effects of matrine in CRC showed that glucose uptake and lactic acid production in HCT116 and SW620 after matrine treatment decreased, inhibiting the Warburg effect in CRC (Hong et al., 2019). Mechanistically, the transcription and expression of HIF-1α and its downstream related proteins GLUT1, HK2, and LDHA are significantly inhibited by matrine treatment, and knockdown of HIF-1α or overexpression of HIF-1α could reverse matrine’s effect on glucose uptake and lactate production (Hong et al., 2019).

#### 5.4.4 Quercetin

Quercetin is a flavonoid extract that is common in fruits and vegetables and which exerts anticancer effects by inhibiting cell proliferation, inducing cell apoptosis, and delaying the invasion and metastasis of cancer cells (Wang et al., 2022; Xia et al., 2022). Quercetin can induce apoptosis of HCT116 cells by inhibiting the activity of AMPK under hypoxia, down-regulating the expression of HIF-1α and its downstream vascular endothelial growth factor and GLUT1 (Kim et al., 2012). A previous study analyzed the effects of quercetin on the transcriptome and protein change in the discretion-rate colon mucosa of F344 rats and found that quercetin downregulated the expressions of glycolysis-related enzymes such as F-1,6-BP, glyceraldehyde-3-phosphate dehydrogenase (GAPDH), ENO1, PK, and upregulated the expression of genes related to mitochondrial fatty acid degradation, such as 3-hydroxy-3-methylglutaryl-CoA synthase 2, acetyl-CoA acyltransferase 1, and acyl-CoA dehydrogenase short-chain (Dihal et al., 2008). Quercetin may transform the glycolysis of CRC into the degradation of mitochondrial fatty acid in F334 rats, causing mitochondrial dysfunction and inhibiting the development of CRC (Dihal et al., 2008).

### 5.5 Phytochemicals targeting c-myc to affect glycolysis in CRC

#### 5.5.1 Diosgenin and dioscin

Diosgenin (DSG) is a bioactive steroidal sapogenin, abundant in fenugreek seeds (Raju and Mehta, 2009). DSG not only showed inhibitory effects on the proliferation, invasion, and metastasis of CRC cells but also induced apoptosis (Li S. Y. et al., 2021). In SW1116 cells, DSG showed inhibitory effects on cancer cells glycolysis, including reduction of ECAR, OCR, lactate production, glucose uptake, and expression of GLUT2, GLUT3, and PC (Li S. Y. et al., 2021). Mechanistically, DSG regulated the expression of proteins involved in apoptosis, migration, invasion, and metabolic phenotypes of CRC cells by inhibiting the cAMP/PKA/CREB pathway in SW1116 and RKO cells. On xenograft tumor model of nude mice with SW1116 cells showed that 30 mg/kg DSG can reduce the weight of tumors *in vivo*, and inhibit the expression of Ki67 and proliferating cell nuclear antigen, Consistent with previous results in cells, DSG also inhibited the cAMP/PKA/CREB signaling pathway in tumor of nude mice (Li S. Y. et al., 2021). The cAMP/PKA/CREB pathway has been confirmed to regulate the growth, migration, invasion, and metabolism of cancer cells, and it is closely related to CRC metastasis (Zhang et al., 2020b; Fujishita et al., 2022).

Dioscin, a structural analog of DSG, is also a steroid saponin isolated from *Dioscoreae rhizome* and *Paridis rhizome,* among others. Dioscin downregulates CRC cells (HT-29, HCT-116, and SW480) proliferation and colony formation by promoting FBW-7-mediated c-myc ubiquitination, leading to the downregulation of c-myc and HK2 expression, reduced lactate consumption and glucose absorption, and inhibition of glycolysis. In xenograft models of HCT116 and HT29, tumor growth was inhibited after 5 mg/kg dioscin every 2 days treatment, and expression of c-myc, Ki-67, and HK2 was significantly reduced, and apoptosis was upregulated (Wu et al., 2020). In addition, Dioscin was shown to downregulate S-phase kinase-associated protein 2 (Skp2) protein levels and inhibit the expression of HK2 and aerobic glycolysis in CRC cells *in vitro* and *in vivo* by inhibiting Skp2 S72 phosphorylation and enhancing Skp2 ubiquitination and degradation in a cadherin 1(Cdh1) dependent manner (Zhou L. et al., 2020). As an oncogene, the Skp2 gene is closely related to the pathogenesis of CRC and is essential for the growth of CRC cells.

#### 5.5.2 Astragalus saponins

Astragalus membranaceus is a well-known Chinese tonic, that is used as an immune stimulant, antioxidant, diuretic, etc. (Fu et al., 2014). Astragalus saponins (AST), are extracted from the medicinal plant *Astragalus membranaceus*. Studies have shown that AST downregulates the mRNA expression and enzyme activities of LDHA and HK2 in SW620 cells, inhibits glucose uptake and lactate production, induces apoptosis, and inhibits cell growth and proliferation (Guo et al., 2019). The authors speculated that this may be related to the downregulation of c-myc expression in SW620 cells by AST. In DSS-induced colitis mice, AST attenuates DSS-induced body weight loss and inflammatory response and downregulated the mRNA expression of c-myc and glycolysis-related enzymes, such as LDHA, GLUT, and HK2, which was consistent with the *in vitro* results (Guo et al., 2019).

#### 5.5.3 Morin and esculetin

Morin is a polyphenol compound originally isolated from members of the Moraceae family such as *mulberry figs* and *fustic* (Chung et al., 2016). Esculetin is the principal bioactive ingredient of *Fraxinus rhynchophylla* Hance (Zhang et al., 2022). In a rat colon cancer model induced by 1,2-dimethylhydrazine (DMH), Morin and esculetin at a dose of 50 mg/kg were shown to reduce the incidence of DMH-induced tumors and down-regulating the expression of related proto-oncogenes. It also affects tumor metabolism through the β-cateinin/c-myc signaling pathway, including inhibiting the protein expression of GLUT, HK2, PKM2, LDHA and other glycolysis-related proteins, and promoting glutaminolysis (Sharma et al., 2017).

Morin also downregulated GLUT1 expression and glucose uptake, inhibited ATP production of SW480 cells, and induced ROS production, affecting mitochondrial function and promoting SW480 cells apoptosis (Sithara et al., 2017).

#### 5.5.4 Apigenin

Apigenin is a natural flavonoid compound, which is common in vegetables and fruits (Sharma et al., 2019) and which significantly restrains cell survival and colony formation of HCT116, HT29, and DLD1 (Shan et al., 2017). Further studies showed that apigenin inhibited the mRNA and protein levels of PKM2 by specifically targeting the allosteric FBP binding site of PKM2 and blocking β-catenin/c-myc/PTBP1 signaling pathway to HCT116 cells, reducing the extracellular acidification rate, glucose consumption, and lactate production, blocking the glycolytic metabolism of HCT116 cells (Shan et al., 2017).

### 5.6 Phytochemicals targeting other pathways that affect CRC glycolysis

#### 5.6.1 Oroxylin A

Oroxylin A (OA) is a flavonoid isolated from the root of *S. baicalensis* Georgi., which exerts various functions including cell growth inhibition and apoptosis induction in various cancer cells (Wei et al., 2013). A previous study showed that OA significantly inhibited glucose uptake and lactate production of HCT116 cells, and it significantly increased the protein level of p53 and the expression of TIGAR in cells and inhibited downstream the mRNA and protein levels of PGM and GLUT4; these inhibitory effects were confirmed to be mediated by p53 (Zhao et al., 2015). Mechanism studies showed that OA by promoting the deacetylation of sirtuin-3(SIRT3), increases the lipid phosphatase activity of PTEN and negatively regulates the transcription of MDM2, thus reducing the degradation of P53 (Zhao et al., 2015). An *in vivo* study showed that 100 mg/kg OA inhibited the growth of nude mice xenograft tumor-inoculated HCT116 cells by down-regulating MDM2 level and glycolytic protein mediated by p53. As one of the widely studied tumor suppressor genes, the p53 gene plays an important role in inhibiting the growth of tumor cells, inducing apoptosis, and regulating metabolism (Komarova and Gudkov, 1998; Vogelstein et al., 2000). SIRT3 is a member of the SIRT family of proteins and has been considered to be related to genomic stability, tumorigenesis, and energy metabolism (Finkel et al., 2009). As a tumor suppressor gene, PTEN has been demonstrated in previous studies to inhibit p53 degradation by controlling MDM2 P1 promoter activity through its lipid phosphatase activity (Freeman et al., 2003).

#### 5.6.2 Ganoderma acid D

*Ganoderma lucidum* is a type of mushroom that grows on plum trees in Asia and is believed to protect body functions, regulate immunity, and promote health and longevity (Jin et al., 2016). Ganoderma acid D (GAD) is a kind of triterpene compound, which is the main active component of *G. lucidum* (Yuan et al., 2022). Studies have shown that GAD can reduce glucose uptake, lactic acid production, pyruvate production, and acetyl COA level of HT29 and SW620 cells by upregulating the expression of SIRT3 protein, inactivating acetylation cyclophilin D (CypD), thus playing a role in regulating CRC energy metabolism (Liu et al., 2018). SIRT3 is a mitochondrial deacetylase, and SIRT3 plays a key role in ROS and limiting cell oxidative damage (Torrens-Mas et al., 2017). SIRT3 can cause cell death under stress conditions, thus acting as a tumor suppressor (Torrens-Mas et al., 2017). CypD is one of the SIRT3-modified target proteins in mitochondria.

#### 5.6.3 Rosmarinic acid

*Rosmarinus officinalis* L. is a plant of the Lamiaceae family native to the Mediterranean region, which has anti-inflammatory, antioxidant, anti-proliferation and other pharmacological effects (de Oliveira et al., 2019). As a water-soluble polyphenol compound, rosmarinic acid (RA) was first isolated from *R. officinalis* L. (Lamiaceae) (Khojasteh et al., 2020). RA inhibits the inflammatory response to the tumor microenvironment by inhibiting the expression of miR-155-5p in HCT8 and HCT116 cells, down-regulating the levels of transcription factor STAT3 and inflammatory factor Interleukin 6(IL-6), resulting in the inhibition of glucose consumption and lactic acid production in CRC cells, and the down-regulating expression of LDH and HIF-1α, and play the role of anti-Warburg effect (Xu et al., 2016). After further intervention with a miR-155-5p agomir drug, it was found that the above inhibitory effects of RA were reversed (Xu et al., 2016). Recent studies have shown that miR-155 plays an important role in immunity, inflammation, cardiovascular disease, and tumors (Elton et al., 2013). IL-6 is an inflammatory molecule that is highly expressed in tumor tissues and closely related to tumor cell proliferation (Kumari et al., 2016).

#### 5.6.4 6,7-dimethoxy-1,2,3,4-tetrahydro-isoquinoline-3-carboxylic acid

The isoquinoline alkaloids 6,7-dimethoxy-1,2,3,4-tetrahydro-isoquinoline-3-carboxylic acid (M1) are isolated from the seeds of *Mucuna pruriens(Kumar et al., 2016)*. The cytotoxicity of M1 was determined by the cell growth inhibition (MTT) method, which showed its antiproliferative activity against Huh-7 (human hepatoma cell line) (Kumar et al., 2016). M1 also shows anticancer effects in CRC. A study showed that M1 at 10 and 25 mg/kg doses for 15 days inhibited the high expression of inflammatory cells such as IL-6 in DMH-induced CRC cells and inhibited the activation of the JAK2/STAT3 pathway mediated by IL-6 (Mishra et al., 2018); further, it downregulated the expression of enzymes such as LDH in CRC liver metastasis and inhibited the high uptake of lactic acid and glucose in CRC cells (Mishra et al., 2018).

## 6 Critical considerations

### 6.1 Potential side-effects of phytochemicals

Although some phytochemicals show specific cytotoxicity to tumor cells, they may elicit adverse effects in humans, depending on the dosage and preparation. Studies have shown that the semi-lethal concentration (LC50) of quercetin is 448.45 ± 0.46 mg/L, and low oral dosages of quercetin (128 mg/kg) do not cause any significant changes in body appearance and general behavior in mice (Pal and Tripathi, 2020). However, higher dosages of quercetin (450 mg/kg) showed mild toxic effects, including weight loss and liver function impairment (Pal and Tripathi, 2020). Oral dosages of quercetin-magnesium were recommended to be restricted to less than 130 mg/kg to avoid possible toxic effects (Ghosh et al., 2017). Andrographolide show toxic side effects in a time-dependent manner, and they can significantly inhibit the proliferation of human renal tubular epithelium cells, induce cell apoptosis and inflammation, and increase nephrotoxicity (Zeng et al., 2022). Meanwhile, it has been reported that with andrographolide (PN355, Paracelsian, Inc.) in a phase-I clinical trial with HIV-positive patients, adverse effects such as anaphylaxis, fatigue, headache, rash, diarrhea, dry mouth, and decreased taste occurred with increasing dosage, and the adverse events disappeared 6 weeks after trial interruption (Calabrese et al., 2000). Determining the safe dosage of phytochemicals *in vivo* and balancing the relationship between their therapeutic effects and toxic side effects is a prominent difficulty in the development of phytochemical drugs.

### 6.2 Bioavailability

Some phytochemicals have been demonstrated to inhibit the glycolytic pathway of CRC *in vitro* and *in vivo* studies, however, due to their poor water solubility, low bioavailability, low cell uptake, etc., their further application in clinical practice is limited (Mohapatra et al., 2022). How to prepare such drugs so that they can be effectively utilized by humans is one of the bottlenecks of drug development. Therefore, one main objective of drug development is to improve the absorption and pharmacokinetics of drugs *in vivo* (Estrela et al., 2017). Piperine, a component of black pepper (Piper spp.), can improve the bioavailability of curcumin, the tea polyphenol (-)-Epigallocatechin-3-gallate, and other phytochemicals by inhibiting their glucuronidation (Lambert et al., 2004; Shaikh et al., 2009).

A further prominent avenue to improve the bioavailability of phytochemical drugs is novel drug delivery systems such as nanocarriers, which can alter the pharmacokinetics and improve the stability and half-life of drugs (Aqil et al., 2013). Quercetin has shown promising anticancer activity in current studies, however, its applicability is limited due to its poor water solubility *in vivo*, poor deliverability, and unstable molecular structure. Nano-conjugated quercetin can overcome the limitations of quercetin and enhance its anticancer effect, thus offering development prospects (Vinayak and Maurya, 2019). Liposome nanocarriers of apigenin can improve its bioavailability, and dual-drug-loaded liposomes with apigenin and 5-FU show higher cytotoxicity, stronger inhibition of angiogenesis and cell proliferation, and increased apoptosis. This delivery mode also showed upregulation of AMPK and downregulation of downstream HIF-1 activity and stronger reversal of the Warburg effect in CRC cells, compared to the single agent (Sen et al., 2019).

### 6.3 Synergistic interactions of phytochemicals with other treatments

The combination of various phytochemicals can improve the blood concentrations and bioavailability of drugs. Therefore, identifying the synergistic effects of drugs is an important aspect of drug development. The synergistic effect of phytochemical drugs combined with chemoradiotherapy is thus a key direction of treatment research. HF and artemisinin (ATS) show synergistic effects in CRC, manifested as synergistic induction of apoptosis and autophagy by HF-ATS (Gong et al., 2022). Quercetin can be used in combination with 5-FU (Boersma et al., 1994), doxorubicin (Atashpour et al., 2015), and other chemotherapy drugs to enhance its anti-cancer effect and to reduce the cytotoxicity of chemotherapy drugs. Furthermore, quercetin also played a significant role in reducing the mechanism of CRC chemoresistance (Zhou Y. et al., 2020). Oroxylin A and wogonin can synergistically inhibit MCF-7 proliferation and induce cell apoptosis, and baicalein also shows increased gastric cancer AGS cells sensitivity to 5-FU by inhibiting glycolytic flux (Cheng et al., 2018). Currently used phytochemicals have shown great potential in the treatment of cancer, and synergistic effects of phytochemicals and other drugs are a key aspect of drug development.

### 6.4 Clinical transformation of phytochemicals

Numerous clinical trials have been conducted to develop phytochemical drugs. In a double-blind, randomized, placebo-controlled trial on berberine and patients who had undergone colorectal polypectomy within 6 months before recruitment, the experimental group received berberine (0.3 g, twice per day) and were followed for 2 years; the results showed that the berberine treatment group had a lower incidence of recurrent adenomas (Chen et al., 2020). As a well-known proprietary Chinese medicine, Kangai injection consists of extracts of Astragalus, Ginseng, and Matrine. In a randomized trial, Kangai injection and platinum combination therapy have been shown to have a greater benefit than chemotherapy alone in patients with advanced non-small cell lung cancer, which can enhance the body’s immunity and reduce the toxicity of chemotherapy drugs (Cheng et al., 2018). Aidi injection plays a role of advantages in the treatment of liver cancer (Liu et al., 2021). In CRC patients, the combined use of FOLFOX with Chinese herbal medicines such as Delisheng and Xiaoaiping shows higher safety and improved body functioning than treatment with FOLFOX alone (Zhang et al., 2019). Further evidence also suggests that the use of chemotherapeutic drugs combined with Chinese patent medicine may be more beneficial, and some studies have shown that phytochemicals have significant anticancer properties, however, further research is needed (Thomasset et al., 2007).

## 7 Summary and prospect

In the past few decades, CRC screening has become increasingly common, and treatment methods have been developed, however, death and incidence rates of CRC are still increasing. At present, there is a lack of effective targeted therapy for CRC. Aerobic glycolysis, as one of the characteristics of tumors, is considered a therapeutic target. It has been found that many malignant tumors, including CRC, have higher glycolysis rates to maintain the proliferation, invasion, and metastasis of cancer cells. In CRC, glycolysis-related enzymes and transporters (e.g., GLUT1, HK2, LDHA, PKM2) are highly expressed, and the upregulation of HIF-1, AMPK, c-Myc, PI3K/Akt/mTOR and other related pathways and transporters also promote the glycolysis process of CRC. This review focuses on phytochemicals that target the glycolytic pathway in CRC. There is *in vitro* and *in vivo* evidence that some phytochemicals can affect the growth, invasion, and metastasis of CRC and induce CRC apoptosis by targeting the glycolytic pathway.

The results compiled for this review were classified according to different mechanism pathways targeted, including phytochemicals targeting PI3K/AKT/mTOR pathway (e.g., Thymoquinone and Daphnetin), AMPK pathway (e.g., Isoliquiritigenin and Resveratrol), HIF-1 (e.g., Worenine and Berberine), and c-myc (e.g., Astragalus saponins and Morin), and some phytochemicals directly affecting the glycolytic pathway of CRC without involving the above pathways. Examples include phytochemicals that directly target GLUT1 (e.g., Scutellarin and Kaempferol), PKM2 (e.g., Anthocyanin and Silybin), and MCT4 (e.g., Epigallocatechin-3-gallate). Although the phytochemicals mentioned above affect CRC cells through different pathways, they all suggest bright prospects for targeting CRC glycolytic pathways in the treatment of CRC.

Tumor metabolism is highly complex. Some phytochemicals have complex and diverse molecular targets, and further experiments are needed to verify their anticancer effects. We introduce several phytochemicals targeting CRC glycolysis metabolic pathways, which appears to be a promising direction; however, further research is required, including on how to maintain the stability of plant chemicals in the body, how to prepare plant chemicals that can be transmitted directly to the lesion site, and how to improve blood concentrations of phytochemicals. Therefore, exploiting synergistic effects of phytochemicals with other drugs may provide new ideas for further research and development of anticancer drugs. In addition to the regulation of glucose metabolism, some phytochemicals have shown the therapeutic action of multi-target and multi-pathway, and their anticancer mechanisms need further verification. Although some phytochemicals have shown potential therapeutic effects on colorectal cancer, but long-term evaluation for their possible toxic and side effects is required. In the future, more researches on phytochemicals targeting glycolysis can provide evidence for the drug development in CRC therapy.

## Figures and Tables

**Table:** 

**AMPK**	Adenosine 5′-monophosphate (AMP)-activated protein kinase
**ALDOA**	Aldolase, fructose-bisphosphate A
**Cdh1**	Cadherin 1
**CamKKB**	Calmodulin-dependent protein kinase beta
**CAFs**	Cancer-associated fibroblasts
**CoA**	Coenzyme A
**CRC**	Colorectal cancer
**cAMP**	cyclic adenosine monophosphate
**CREB**	Cyclic-AMP response binding protein
**CypD**	Cyclophilin D
**DMH**	1,2-dimethylhydrazine
**ENO1**	Enolase 1
**EGFR**	Epidermal growth factor receptor
**ECAR**	Extracellular acidification rate
**ERK (1/2)**	Extracellular regulated protein kinases (1/2)
**5-FU**	5-Fluorouracil
**GLUTs**	Glucose transporters
**GAPDH**	Glyceraldehyde-3-phosphate dehydrogenase
**hnRNPA1**	heterogeneous nuclear ribonucleoprotein A1
**HK(2)**	Hexokinase (2)
**HIF-1(α)**	Hypoxia-inducible factor-1(α)
**IL-6**	Interleukin 6
**JAK2/STAT3**	Janus kinase 2/signal transducer and activator of transcription 3
**LDH (A/B)**	Lactate dehydrogenase (A/B);
**LncRNA GLCC1**	glycolysis-associated lncRNA of colorectal cancer
**LncRNAs**	Long non-coding RNAs
**mTOR**	mammalian target of rapamycin
**MCTs**	Monocarboxylic acid transporters
**OCR**	Oxygen consumption rates
**PTEN**	Phosphatase and tensin homolog
**PI3K/AKT**	Phosphatidylinositol 3-kinases/protein kinase B
**PGM**	Phosphoglucomutase
**PTBP1**	Polypyrimidine tract-binding protein 1
**PKA**	Protein kinase A
**PDK1**	Pyruvate dehydrogenase kinase 1
**PK(M2)**	Pyruvate kinase (M2)
**ROS**	Reactive oxygen species
**SIRT3**	Sirtuin-3
**Skp2**	S-phase kinase associated protein 2
**SOD2**	Superoxide dismutase 2
**TIGAR**	TP53-induced glycolysis and apoptosis regulator
**YAP1**	Yes-associated protein 1
